# Music and Lyric Characteristics of Popular Dutch Funeral Songs

**DOI:** 10.1177/00302228221075471

**Published:** 2022-02-23

**Authors:** Waldie E. Hanser, Ruth E. Mark, Ad J. J. M. Vingerhoets

**Affiliations:** 1 Department of Medical and Clinical Psychology, 7899 Tilburg University, The Netherlands; 2 Department of Cognitive Neuropsychology, 7899 Tilburg University, The Netherlands

**Keywords:** funeral music, linguistic inquiry and word count, linguistic analysis, lyrics, music characteristics

## Abstract

This study compared the characteristics of 150 songs (Dutch lyrics, *N* = 47, English lyrics, *N* = 103), popular at Dutch funerals, to an equal number of non-funeral songs. The variables explored included those linked with the music (valence, energy, danceability, acousticness, key, and tempo); and lyrics, namely: linguistics-related (first-person singular/plural, second-person pronouns; past, present, future tense; expressed emotion (positive, negative words, and the discrete emotional categories anger, anxiety, sadness); and category words (those relating to family, friends, death, religion). Funeral music was lower in valence, energy, and danceability and higher in acousticness than non-funeral music. Furthermore, English funeral music lyrics contained more second-person pronouns and were more future-focused than comparison songs. Funeral lyrics were not particularly negative, but English texts contained more words relating to sadness. In conclusion, funeral music differs in severable notable respects from general popular songs that may reflect the special purpose of this music.

Music is an inextricable part of current Dutch funerals. Since the end of the 20^th^ century, the selection of music played during cremations in the Netherlands has become increasingly personalized and thus more diverse (Bruin-Mollenhorst & Hoondert, 2018; Bruin-Mollenhorst, 2020). This development is partly due to technical improvements in crematoria that enable them to meet personal music requests by the deceased and bereaved more easily than before when standard sets of songs were more commonplace (Bruin-Mollenhorst & Hoondert, 2018). Music selection is currently a standard part of most funeral planning. Funeral homes offer advice on how and what music to select and even provide playlists and multimedia links on their websites as examples. The bereaved can experience the music selection process, sometimes after the wishes of or in agreement with the deceased, as comforting (Viper et al., 2020).

Funeral music may serve different purposes during the ceremony. Bruin-Mollenhorst (2020) distinguishes the following (1) those that serve the ceremony itself (background music when entering or leaving the ceremony room, or while watching pictures of the deceased), (2) music in between speeches that illustrates or empowers their contents, (3) music that gives expression to the identity of the deceased (the favorite music of the deceased; music that represents aspects of the relationship of the bereaved to the deceased or aspects of the deceased’s identity), (4) emotional control and evoking emotions (music that allows the bereaved to give in to their emotions, or to regain their composure), and (5) lastly, music that we listen to without any discernible objectives. These goals fit in well with general psychological functions of music (Schäfer et al., 2013), more precisely: music listening (1) as self-awareness (e.g., music expresses or confirms some part of one’s identity), (2) to bolster social relatedness (e.g., to feel connected with other people), (3) as mood and emotion regulation (e.g., to feel relaxed).

Most music in modern funerals in some way represents the deceased and honors their memory. In these cases, it serves as a musical eulogy (Bruin-Mollenhorst, 2020). Bruin-Mollenhorst (2020) argues that, although lyrics may play a role in the music selection process, for the musical praise, the song texts are often less important than the song as a whole and even less so than the way the entire song connects to the deceased. Contrary to speeches, popular song lyrics are not written explicitly for the deceased, implying that, at most, only some words, a mere sentence, the title, or nothing of the lyrics at all may directly associate with the deceased (Bruin-Mollenhorst, 2020). This does, however, not exclude the possibility that there are recurring words, linguistic properties, or themes that are common in the lyrics of popular funeral music that may make some songs particularly suited to serve this function. Furthermore, it does not explain why certain songs are, despite the increasingly personalized nature of funerals (Bruin-Mollenhorst & Hoondert, 2018), used quite frequently during funerals. In addition to investigating musical aspects, a linguistic analysis of the song texts may reveal why some songs more often serve as funeral music than others. The aim of the current study is twofold, (1) we seek to replicate and expand upon previous findings on the characteristics of funeral music, and (2) we analyze the lyrics of a selection of funeral songs popular in the Netherlands.

## Music Characteristics and Lyrics

Recently, Mollenhorst and colleagues (2016) examined music characteristics (valence, energy, and tempo) of often-used songs in Dutch crematoria. Based on data from audio analyses, funeral music (*N* = 3703 songs) was shown to be lower in valence, tempo, and energy than two popular comparison playlists (Top 40 and Top 2000). This resulted in the characterization of funeral music as solemn, serene, and tender. It is, however, unclear which specific parts of a song evoke these emotions since these investigators considered the songs as a whole and did not study the lyrics separately. Analyzing the song as a whole may make sense because, as a listener, it is difficult to separate the impact of the melody from the lyrics. However, considering song texts separately may also prove valuable since the lyrics can substantially contribute to the emotional experience of music and are a way for listeners to relate to songs.

Previous studies have yielded mixed results regarding the question as to whether the melody or the song text is mainly responsible for the emotions reported while listening to music (see Ali & Peynircioğlu, 2006 for an overview). However, the research findings of these authors suggested that a song’s melody is the dominant determinant, though lyrics can contribute substantially to the emotions perceived in the song. More specifically, they demonstrated that emotion in response to sad and angry music accompanied by emotionally matching lyrics is more intense. In contrast, in the case of happy and calm music, emotionally congruent lyrics may reduce the intensity of the feelings. Brattico and colleagues (2011) replicated the finding that happy music without lyrics results in stronger positive emotions than happy music with lyrics. In an fMRI study, Brattico et al. (2011) further found more extensive brain activation, particularly in the limbic system, when listening to sad music with lyrics as opposed to without, and happy music without as opposed to with lyrics. Lyrics seem to play a more prominent role when listening to sad music, while music characteristics may be of greater importance when listening to happy music. Lastly, the processing of lyrics may be deeper when accompanied by sad music than in the case of happy music (Fiveash & Luck, 2016).

Emotionally congruent lyrics thus intensify sad music, while the opposite is true for happy music. This intensification may partly explain the attraction most people have for sad music for mood-regulatory purposes (e.g., Garrido, 2017; Van den Tol & Edwards, 2013; Van den Tol, 2016). Individuals apply mood regulation techniques to improve their moods. When employing music for this purpose, listeners typically prefer music that best reflects the mood they are in (e.g., Van den Tol, 2016). It is mainly the lyrics of sad music that help listeners to relate and connect to it (Eerola et al., 2015; Van den Tol & Edwards, 2013; Van den Tol, 2016). In general, lyrics may thus be particularly salient to the listeners’ mood, and they may strongly reflect the situation the music is heard in (Baltazar et al., 2019). Funeral music’s lower valence, energy, and tempo than popular music (Mollenhorst et al., 2016) suggests it will sound *sad* (Schellenberg & Von Scheve, 2012). Funeral music lyrics may follow this trend and contain more negative words and more words that express sadness.

Several recent survey and interview studies further exemplify the importance of lyrics to mood regulation through music and specific peak emotional experiences. Song texts are particularly relevant to listeners who seek consolation through music, although the melody and music characteristics are more important (Hanser et al., 2016; Saarikallio & Erikkilä, 2007; Ter Bogt et al., 2017). Lyrics further play a role in the experience of chills (Bannister, 2020) and crying over music (Cotter et al., 2019; Hanser et al., 2021), especially when negative emotions accompany the crying episodes (Cotter et al., 2019). This further exemplifies a connection between sadness and the relevance of lyrics. Personal meaning or memories triggered by a song may contribute more to crying over music than its music characteristics or its lyrics separately (Cotter et al., 2019; Hanser et al., 2021). The studies mentioned above, however, did not investigate the properties of song texts.

## Linguistic Properties and Lyrical Content of Popular Music

Song texts thus certainly play a significant role in the emotions evoked by and perceived in music. However, their linguistic properties and content have received little attention from researchers, particularly when considering specific situations, such as funeral rites, or for personal uses like mood regulation. Yet, an analysis of happy and sad song texts with Linguistic Inquiry and Word Count software (LIWC; Pennebaker et al., 2015) revealed several intriguing differences. More precisely, sad songs contained more negatively valenced words, particularly expressions of sadness and anger, while happy songs included more positively valenced words. Further, happy songs had more words in the present tense than sad songs (Garrido, 2017, p. 24–25).

The use of specific personal pronouns may be of further relevance to the (social) functions of popular music lyrics. Specifically, more second-person pronouns (you, yours) have been linked to increased song liking and commercial success, primarily when these second-person pronouns refer to “you” as the object of a sentence (Packard & Berger, 2020). The underlying idea is that “you” makes listeners think of a person significant to them, which may, in turn, help lyrics bolster social relatedness (Packard & Berger, 2020). Alternatively, the use of first-person singular pronouns (I, me, mine) in lyrics and personal writing is associated with a focus on the self, while first-person plural pronouns (we, us) are an indicator of emphasis on social relationships (DeWall et al., 2011; Rude et al., 2004; Tausczik & Pennebaker, 2010). DeWall and colleagues (2011) showed an increase in first-person singular pronouns, a decrease in first-person plural pronouns, and a reduction in positive and social words in lyrics between 1980 and 2007. This finding suggests that song texts became more individualistic and less positive as popular music evolved. First-person plural pronouns are, however, in general not used very often in song texts of popular music (DeWall et al., 2011; Packard & Berger, 2020).

Thematically, the lyrics of most popular music deal with romantic relationships (Christenson et al., 2019), while only roughly 10% of songs cover topics surrounding family and friends. Songs that explicitly mention death and dying are rare, although these topics are dealt with more frequently as popular music evolves. Funeral music is likely to contain references to romantic relationships or family and friends.

## The Present Study

The present study looks at both the music and linguistic characteristics of frequently used songs during Dutch funerals. We compare the properties of funeral song texts to a control selection that consists of popular songs by the same artists. To this end, we formulated the following research questions and related hypotheses:

1. The first focus is on music characteristics. We expect to replicate findings by Mollenhorst et al. (2016) on music characteristics. We thus expect to find lower valence, energy, tempo, and danceability values and higher values of acousticness for funeral music than for control songs. Funeral music will further consist of more songs in major than minor key. These values on music characteristics correspond more to sad than to happy music.

We additionally analyzed the lyrics. Regarding linguistical properties, we assume lyrics of funeral music to resemble lyrics of sad music. Given the social role of music during funerals, we further expect pronouns, temporal focus, and specific topics to reflect this function.

2. Funeral music contains more negative and less positive words than control songs. Funeral music further focuses less on the present and more on the past than control music.
3. We hypothesize that funeral music contains fewer first-person singular pronouns and more first-person plural and second-person pronouns than control songs. Family and friends, as well as topics surrounding death and religion, are expected to be mentioned more often in funeral songs.

Lastly, since popular music often focuses on romantic relationships, we anticipate frequent words related to this subject. We have no reason to expect a difference between these words for funeral or control songs.

## Method

### Materials

#### Song Selection and Lyrics

The selection of songs was based on lists of frequently used songs during funerals, burials, and other farewell rites. These lists are from Dutch websites^1^ of (insurance) companies (Dela, 2021; RVS Grafmonumenten, 2021; Uitvaartverzekering Vergelijken Loont, 2021) that provide services to organize or finance funerals. We consulted these websites in 2017, 2020, and 2021. We limited our selection to songs with Dutch or English lyrics.

The control selection contained songs by the same artists as the funeral song selection. This selection was based on a song’s popularity on Spotify: we picked (according to Spotify) the most popular song by the same artist. If this piece of music was also present in the funeral selection, the second-most popular was chosen. We have attempted to always use a target and a control song by the original artist in the case of cover songs. Our primary focus was on English songs since they formed a more significant part of the popular funeral songs than Dutch ones. The complete selection consisted of 206 songs with lyrics in English (103 funeral and 103 control) and 94 songs with lyrics in Dutch (47 funeral and 47 control).

We gathered the song lyrics from Genius.com (Genius, 2021) or comparable lyric depositories if the texts were not available from this website (e.g., Muzikeum.eu; Muzikum, 2021). Texts were manually spell-checked and prepared for analyses through LIWC and *tidytext* (e.g., *goin’* was changed into *going*). The total number of words was: English funeral 22098, English control 26563 (Total 48661), Dutch funeral 11531, Dutch control 12445 (Total 23976)

#### Software

##### Music Characteristics

Data on music characteristics were collected from the Spotify website (Spotify for Developers, 2021) through the Application Program Interface (API). Similar to the Mollenhorst and colleagues (2016) data, these audio analyses consider songs as a whole. The API was accessed through the R-package *spotifyR* (Thompson et al., 2017). We collected information on a track’s acousticness (‘a measure from 0.0 to 1.0 of the degree to which the track is acoustic’), danceability (‘a value from 0.0 to 1.0 that measures how danceable a track is based on several factors, including tempo, rhythm, beat’), energy (‘perceptual measure from 0.0 to 1.0 on how intense and active a track is'), tempo (‘the overall estimated track tempo in beats per minute (BPM)’), mode (‘track modality 0 = minor, 1 = major’), and valence (‘measure from 0.0 to 1.0 on musical positiveness conveyed by a track’) (Spotify for Developers, 2021).

##### Lyrical Properties

Lyrics were analyzed using the Linguistic Inquiry and Word Count software (LIWC2015; Pennebaker et al., 2015). LIWC counts words based on classifications in a dictionary with reliable psychometric properties and returns these values as percentages of the word total (Pennebaker et al., 2015). This program has been used before in the analyses of song lyrics (e.g., DeWall et al., 2011; Garrido, 2017; Packard & Berger, 2020; Petrie et al., 2008). We used the in-built English dictionary to analyze English lyrics (Pennebaker et al., 2015), whereas Dutch lyrics were assessed with the available Dutch dictionary (Boot et al., 2017). Due to these dictionary differences, we considered Dutch and English songs separately. The word categories we investigated were: pronouns (first-person singular/plural, second-person), temporal orientation (past, present, future), emotional valence (positive, negative), discrete emotional categories (anger, anxiety, sadness), and specific topics (friends, family, death, religion).

We explored word sentiment with the R-package *tidytext* (Silge & Robinson, 2016) and the NRC Word-Emotion Association Lexicon (Mohammad & Turney, 2013). *Tidytext* assigns words into emotional categories based on a lexicon and returns these values as word frequencies. Our focus with *tidytext* is on words responsible for positive and negative affect.

#### Statistical Analyses

We performed a series of independent sample t-tests to look for differences between the two selections. We additionally report the effect-size Cohen’s *d* (small 0.2, medium 0.5, and large 0.8; Cohen, 1988). Analyses were carried out using IBM SPSS 23.0 and R 4.0.4 (R Core Team, 2021).

## Results

### Music Characteristics

The analyses of the music characteristics (see Table 1) revealed several significant differences between funeral music and control songs. Valence, energy, and danceability were significantly lower for the funeral songs than for the control selection. Funeral songs were also more acoustic and more often in a major key than control songs. The effect-sizes varied from medium to large. There were no statistically significant differences in tempo.

**Table 1.** Overview of the music characteristics of funeral and control songs.

	Funeral	Control	*t*	df	*p*	Cohen’s *d*
English	*N* = 103	*N* = 103				
Music characteristics						
Valence *	0.28 (.16)	0.54 (0.24)	−9.19	177.56	<.001	−1.28
Energy*	0.39 (0.18)	0.56 (0.24)	−5.85	192.11	<.001	−0.82
Tempo (BPM)	113 (29.10)	118 (26.23)	−1.44	201.84	.151	−0.20
Acousticness*	0.51 (0.32)	0.34 (0.30)	4.00	203.58	<.001	0.56
Danceability*	0.44 (0.14)	0.55 (0.15)	−5.22	200.89	<.001	−0.73
Key: % Major key	85.44%	69.90%				
	Funeral	Control	*t*	df	*p*	Cohen’s *d*
Dutch	*N* = 47	*N* = 47				
Music characteristics						
Valence*	0.34 (0.18)	0.57 (0.27)	−4.90	81.02	<.001	−1.01
Energy*	0.40 (0.20)	0.52 (0.22)	−2.74	90.82	.007	−0.57
Tempo (BPM)	114 (29.47)	116 (30.81)	−0.33	91.82	.739	−0.07
Acousticness	0.51 (0.32)	0.47 (0.28)	0.72	90.41	.474	0.15
Danceability*	0.51 (0.12)	0.57 (0.13)	−2.50	90.50	.014	−0.52
Key: % Major key	83.98%	85.11%				

*Note*. 1) The music characteristics except for tempo and key have a range from 0 – 1, 2) BPM = beats per minute, 3) Mean values, SD given in parentheses, 3) Cohen’s *d*: small 0.2, medium 0.5, and large 0.8.

Music characteristics of the Dutch selection followed a similar pattern as the English-sung tracks. However, most effects were smaller: Dutch funeral songs also scored lower on valence, energy, and danceability. Acousticness and tempo did not differ significantly, although a similar pattern as in the English selection with funeral music being more acoustic was observed. There was no difference between the distribution of major and minor key for Dutch songs.

### Analyses of Lyrics with LIWC

We investigated the total word count, pronouns, sentiment, temporal focus, social connections, and topics using the LIWC software (Tables 2 and 3). Funeral lyrics in both English and Dutch songs contained fewer words than the control songs, but this difference was only significant for the English songs. In both languages, there was substantial variability in lyric length. Moreover, most personal pronouns were first-person singular, followed by second-person pronouns. First-person plural pronouns were rare. First-person singular and plural pronouns did not differ significantly between selections. In contrast, second-person pronouns were significantly more common in English funeral lyrics compared to control songs. A comparable, non-significant pattern was present for Dutch lyrics.

**Table 2.** Overview of the results of the linguistic inquiry and word count-analyses of english funeral and control lyrics.

	Funeral	Control	*t*	df	*p*	Cohen’s *d*
Words per song*	215 (80.24)	258 (110.38)	−3.22	186.27	.001	−0.45
Pronouns						
First-person singular	8.54 (5.39)	8.61 (5.09)	−0.11	203.29	.915	−0.01
First-person plural	1.25 (2.06)	0.93 (1.58)	1.27	191.42	.206	0.18
Second-person*	6.08 (4.28)	4.91 (3.41)	2.18	194.46	.031	0.30
Emotion						
Negative emotion	2.11 (1.77)	2.12 (2.49)	−0.02	184.33	.987	0.00
Positive emotion	4.57 (2.91)	4.24 (3.06)	0.79	203.58	.428	0.11
Anger	0.30 (0.58)	0.47 (1.11)	−1.38	154.13	.170	−0.19
Anxiety	0.20 (0.42)	0.33 (0.94)	−1.27	141.64	.205	−0.18
Sadness*	1.14 (1.43)	0.74 (1.10)	2.28	191.84	.024	0.32
Time orientation						
Focus past	3.27 (3.06)	3.66 (3.38)	−0.85	202.02	.395	−0.12
Focus present	15.05 (5.07)	15.11 (4.96)	−0.09	203.90	.930	−0.01
Focus future*	2.38 (2.36)	1.79 (1.70)	2.06	185.31	.040	0.29
Social						
Friends	0.34 (0.90)	0.25 (0.47)	0.95	153.35	.345	0.13
Family*	0.18 (0.43)	0.67 (1.65)	−2.93	115.48	.004	−0.41
Topics						
Death	0.24 (0.45)	0.35 (0.89)	−1.14	151.10	.256	−0.16
Religion	0.55 (1.06)	0.71 (1.46)	−0.91	186.67	.366	−0.13

*Note*. 1) Mean values, SD given in parentheses, 2) Word categories are percentages of total, 3) Cohen’s *d*: small 0.2, medium 0.5, and large 0.8.

**Table 3.** Overview of the results of the linguistic inquiry and word count-analyses of Dutch funeral and control lyrics.

	Funeral	Control	*T*	df	*p*	Cohen’s *d*
Words per song	245 (83.33)	265 (82.05)	−1.14	91.98	.257	−0.24
Pronouns						
First-person singular	8.12 (5.43)	7.09 (5.19)	0.94	91.82	.351	0.19
First-person plural	0.84 (1.71)	0.61 (.1.12)	0.76	79.23	.452	0.16
Second-person	6.15 (3.81)	5.59 (4.21)	0.68	91.10	.499	0.14
Emotion						
Negative emotion	2.03 (1.63)	2.00 (1.83)	0.08	90.85	.933	0.02
Positive emotion	2.87 (1.90)	3.70 (2.25)	−1.93	89.50	.057	−0.40
Anger	0.47 (0.86)	0.33 (0.70)	0.84	88.40	.405	0.17
Anxiety	0.29 (0.53)	0.15 (0.28)	1.55	69.67	.126	0.32
Sadness	1.42 (1.34)	1.65 (1.54)	−0.77	90.34	.443	−0.16
Time orientation						
Focus past	2.67 (2.44)	2.81 (3.13)	−0.25	86.91	.806	−0.05
Focus present	11.37 (3.88)	10.95 (3.39)	0.56	90.35	.577	0.12
Focus future*	1.21 (1.12)	0.77 (0.99)	2.04	90.54	.045	0.42
Social						
Friends	0.22 (0.50)	0.31 (0.91)	−0.60	71.80	.552	−0.12
Family	0.45 (0.98)	0.32 (0.63)	0.74	78.82	.460	0.15
Topics						
Death	0.26 (0.59)	0.10 (0.21)	1.73	57.93	.090	0.36
Religion*	0.31 (0.55)	0.10 (0.29)	2.35	69.78	.022	0.48

*Note*. 1) Mean values, *SD* given in parentheses, 2) Word categories are percentages of total, 3) Cohen’s *d*: small 0.2, medium 0.5, and large 0.8.

Positive words were more frequent than negative words for both languages in both funeral and control songs. Words specifically related to discrete categories of anger, anxiety, and sadness were uncommon. English funeral lyrics contained significantly more words related to sadness than controls. Dutch funeral lyrics showed a trend towards less positive emotion words compared to Dutch control lyrics. Overall, Dutch lyrics were less, albeit not significantly, positive and contained more words in discrete negative emotion categories than the English selection.

Regarding temporal focus, lyrics in both selections focused on the present with fewer words relating to the past or future. Nevertheless, both Dutch and English funeral lyrics contained more words associated with the future than control songs. However, it should be noted that the English 2015 dictionary (Pennebaker et al., 2015) uses a slightly different algorithm than the Dutch 2007-dictionary to determine words related to temporal perspectives. This may partly explain the fewer overall words related to temporal focus in Dutch song texts.

Words referring to family and friends, as well as words related to the topics of religion and death, were rare for both funeral and control songs in both languages. On the other hand, there were significantly more family-related words in English control songs than in the funeral lyrics. Dutch funeral lyrics contained significantly more words related to religion than controls, and there was a similar, although non-significant, trend for death-related words. Significant effects for word categories were small to medium in size.

### Analyses of Lyrics with Tidytext

Lastly, we investigated which words contributed to lyric sentiment in the English funeral and control selection only (Table 4) through the R-package *tidytext* with the NRC Lexicon. As in the LIWC-analyses, positive words were more prevalent than negative words for both funeral and control. Positive words reflected more warmth and positivity, while negative words were colder and expressed loneliness. Although *Love* is the most common positive word in both conditions, it occurred more frequently in the funeral songs. When combined with the derived *loving*, it makes up over 25% in the funeral songs instead of 16% in the control songs.

**Table 4.** Most frequently used positive and negative words in funeral and control lyrics.

Funeral				Control			
Positive		Negative		Positive		Negative	
*N* = 1147	*N*	*N =* 642	*N*	*N* = 1464	*N*	*N* = 921	*N*
Love	252	Words	33	Love	207	Leave	30
Sun	53	Feeling	31	Baby	123	Lord	27
Loving	37	Cry	20	Dance	71	Words	23
Feeling	31	Lonely	20	Good	43	Feeling	22
Baby	27	Leave	19	Alive	32	Pain	20
True	26	Cold	15	Master	29	Blind	19
Star	21	Darkness	14	Lord	27	Cry	17
Darling	20	Fall	14	Sun	27	Dust	17
Word	20	Lost	13	Loving	26	Alien	16
Good	19	Wait	13	Beautiful	25	Lonely	16

## Discussion

This study aimed to gain insight into the music and linguistical characteristics of songs popular at Dutch funeral rites. To this end, we explored and compared music and linguistic features of funeral songs and popular control songs by the same artist. To our knowledge, there has been no previous research into the linguistic characteristics of funeral songs. Nevertheless, based on investigations into the song texts of English popular music in general, we expected the use of pronouns in funeral lyrics to show a focus on others and less so on the self (DeWall et al., 2011; Packard & Berger, 2020; Rude et al., 2004; Tausczik & Pennebaker, 2010). We further hypothesized them to be negative, less positive, and less focused on the present (Garrido, 2017) and to contain more words concerning relationships and death-related topics (Christenson et al., 2019).

As expected, funeral songs scored lower on valence and energy, and there was a trend for them to be lower in tempo than control songs. They were also often in a major key. These results corroborate previous findings on funeral music characteristics (Mollenhorst et al.,2016)^2^, thus confirming funeral music’s characterization as sounding solemn, serene, and tender. Moreover, funeral music scored higher on acousticness and lower on danceability than control songs. The higher acousticness emphasizes vocals and lyrics (for example, Eric Clapton’s Tears in Heaven). Differences in these measures were less substantial for Dutch songs compared to English ones. Future work may wish to consider if cultural differences contribute to this discrepancy.

The findings further support our hypothesis that funeral lyrics are more social and sadder than control song lyrics. Regarding pronouns, first-person pronouns (I, me, mine) were the most common personal pronouns in funeral lyrics, but their frequency is not exceptionally high compared to findings in popular music in general (DeWall et al., 2011, p. 202). Given the frequent use of second-person pronouns (you, yours; Packard & Berger, 2020), it can be concluded that funeral lyrics are, as expected, more focused on others than on the self. This was particularly the case for English funeral songs, which contained more second-person pronouns than the artist-matched control songs. As stated before, frequent use of second-person pronouns has been linked to an increase in song liking and commercial success (Packard & Berger, 2020), particularly when the second-person pronoun serves as the object in a sentence (e.g., “Still loving you”). In these cases, it is suggested that listeners do not interpret the content of the lyrics as addressed to themselves by the artist. Instead, they transfer this lyrical content from themselves to a significant other (Packard & Berger, 2020). This seems particularly relevant for funeral music, in which the lyrics may serve as final words or as a farewell from the bereaved to the deceased. Here, song texts may help give voice to what the bereaved are feeling and empower them. Interestingly, this transference does not seem to be limited to sentences containing second-person pronouns but may also apply to sentences with first-person singular pronouns, as exemplified in qualitative research by Viper and colleagues (2020, p. 11): listeners may apply these first-person singular song texts to their own situation, or the lyrics in first-person become more likely essential when sung by a significant other. Additional research may also consider if the bereaved experience parts of the song lyrics as addressed to them when the music was chosen by the deceased and was meant to reflect the relationship with them.

Lastly, as in previous research on popular music lyrics in general (DeWall et al., 2011; Packard & Berger, 2020), first-person plural pronouns (we, us) are less common. The frequency of these words in our funeral selections did not differ significantly from the frequency in the control songs, although a non-significant trend was observed. Interestingly, we found less use of first-person singular pronouns in Dutch control songs than in funeral lyrics, but sources for comparison are unavailable. The pattern for first-person plural and second-person pronouns in Dutch lyrics is like English lyrics but less substantial for second-person pronouns.

The music characteristics of the funeral songs (i.e., low valence and energy) point towards funeral music sounding “more negative.” Nevertheless, we failed to find substantial differences in the prevalence of negative words between funeral songs and controls, and words of discrete emotional categories of anger and anxiety were rare. Words of the sadness category were significantly more frequent in English funeral lyrics than in controls, thus supporting the idea that funeral lyrics express more sadness. Both English and Dutch selections contained more positive words than negative ones, although, in Dutch, positive words were less frequent in the funeral than in the control selection. Overall, the Dutch lyrics were somewhat less positive and contained more words in the anger, anxiety, and sadness categories, pointing to potential differences in the selection process of songs in a native and foreign language. The emotional valence and discrete emotional categories of lyrics, and thus the song texts themselves, may matter more when choosing funeral music in a native tongue. Cultural differences may further play a role and deserve further study.

The current results on the emotional word categories thus do not fully match previous research findings on lyrics of happy and sad music (Garrido, 2017): funeral lyrics are not necessarily emotionally negative lyrics. Further support for this conclusion comes from the temporal focus of the song texts. We found no difference in focus on the past between funeral and control songs. While, overall, there was a greater reference to the past than to the future in general and an even greater focus on the present, we instead found that funeral lyrics, both in English and Dutch, focused more on the future than their artist-matched controls. Examples of this future focus, according to LIWC, are “I will always love you,” “You’ll never walk alone,” and “We’ll meet again.” These examples combine our findings on first and second-person pronouns with a future focus, and further demonstrate the potential social-bonding nature of song texts. We speculate that these parts of lyrics, which are likely to be found in the song’s title and chorus, convey strong emotional messages that may be central as to why some songs are more suitable as funeral music than others. This connects with Bruin-Mollenhorst’s concept of the musical eulogy (2020), in which only part of the lyrics may apply to the deceased or a funeral setting. We further suggest that the emphasis and repetitive nature of these messages as chorus may play an additional role in the choice for these songs. Future research should look further into the musical and linguistic properties of a song’s chorus, specifically in relation to specific uses. Future studies may also wish to consider the meaning of this future focus within the funeral setting: does it, for instance, provide listeners with hope of an afterlife, or does it perhaps help them come to terms with the finality of death?

Lastly, references to death, religion, and friends or family were rare. This could be anticipated based on the thematic analyses of popular songs by Christenson and colleagues (2019). There are not many songs that explicitly deal with these topics. Romantic relationships are, by far, the most common theme in popular music, and words responsible for the affective quality of both English funeral and control songs dealt with this topic. Explicit mentions of “love” (we made no distinction between love as a noun or verb) or a derivative are the most common in both selections, however, more so for funeral lyrics. Follow-up analyses should determine if there is a difference in the type of love, for instance, love of a more spiritual, emotional, or physical nature.

To conclude, we found considerable differences in music and linguistic characteristics of funeral and non-funeral music that may contribute to the choice of these songs for this specific function. Differences in music properties are more substantial than those for linguistic features. It may thus be easier to relate music characteristics to specific uses and functions of music than features of the lyrics.

### Limitations

Several critical comments can be made regarding the applied method. Word count software does not look at the context of words or specific relationships between them. We further only considered some specific word categories, while others may also be important, for instance, words relating to cognitive processes (“never,” “always”), and questions (i.e., “Who wants to live forever?”). Future studies may wish to focus on other categories and word relationships.

For our selection of control songs, we depended on their availability on Spotify. A possible issue of concern regards the values of the music characteristics since it is not clear how the results provided by Spotify for Developers (2021), such as danceability, valence, and acousticness, are compiled (see Howlin & Rooney, 2020 for a discussion), stressing the need for more empirical research into their construct validity. Other studies that have used these measures have, however, found differences and patterns that correspond to what one might expect given previous knowledge on music and its uses (e.g., Baltazar et al., 2019; Baltazar & Västfjäll, 2020; Howlin & Rooney, 2020; Vidas et al., 2021).

A further limitation is our small sample size, especially of Dutch lyrics. Word count software becomes more reliable when more data are available (Pennebaker et al., 2015). We focused on the most frequently used funeral songs instead of on a full funeral home songbook. A full songbook would provide more songs, but it is unclear how popular these songs are as funeral music in general. Including more songs may reveal more differences between funeral songs and (artist-matched) controls. Mollenhorst and colleagues (2016), for example, used a funeral home songbook and compared this to the Dutch Top 2000 and Top 40 popular song lists. It appeared that many of the most frequently used funeral songs, as well as of the control songs were also included in the annual editions of the Dutch Top 2000. Specific artists, music characteristics, as well as linguistic properties, may make these songs particularly popular for multiple functions. This may explain some of the minor differences in terms of linguistic categories. However, this can also be considered a strength of our current study; our selection of popular songs showed apparent differences depending on their specific use. This bolsters the idea that musical and linguistic features also contribute to this choice despite idiosyncratic listener preferences to use songs for particular functions.

Lastly, in the present work, we considered funeral music as a homogenous category of songs. Still, there is sufficient reason to assume that subcategories exist that may depend on, for example, the preference of the deceased, the bereaved, the moment the music is played during the ceremony, culture, as well as various specifics related to the deceased such as their age, relationship to the bereaved and more. Future studies should investigate these more detailed subcategories further.

In conclusion, the current study confirms and expands the modest present knowledge on the characteristics of funeral music based on musical and textual features. The terms solemn, serene, and tender are indeed the keywords to describe the musical properties of this music. Specific word categories may also play a role in determining the utility of songs to serve as funeral music. However, the role of personal preferences and musical features may be more significant than the role of lyrics. Our findings on lyrics underscore their importance in expressing social connections with others (Schäfer et al., 2013). The current study stresses the importance of considering musical, linguistic, and personal properties when relating music to specific (emotional) uses and functions. As such, we hope that our study stimulates other researchers to continue this line of research to achieve a greater understanding of the role of music in funeral rites and the possible determining mechanisms.

## Notes

1. We refer to these specific webpages, but similar list(s) can be found on comparable Dutch websites.

2. Mollenhorst et al. (2016) used the Echo Nest software for their research, which is currently owned by Spotify.
